# Report of the 24th Meeting on Signal Transduction 2021

**DOI:** 10.3390/ijms23042015

**Published:** 2022-02-11

**Authors:** Bastian Schirmer, Klaudia Giehl, Katharina F. Kubatzky

**Affiliations:** 1 Institut für Pharmakologie, Medizinische Hochschule Hannover, Carl-Neuberg-Str. 1, 30625 Hannover, Germany; schirmer.bastian@mh-hannover.de; 2 Signaltransduktion Zellulärer Motilität, Innere Medizin V, Justus-Liebig-Universität Giessen, Aulweg 128, 35392 Giessen, Germany; klaudia.giehl@innere.med.uni-giessen.de; 3 Zentrum für Infektiologie, Medizinische Mikrobiologie und Hygiene, Universitätsklinikum Heidelberg, Im Neuenheimer Feld 324, 69120 Heidelberg, Germany

**Keywords:** signal transduction, STS, conference report, receptor signaling, drug development, infection and inflammation, growth factors, cytokines, immune cell signaling, cancer research, tumor biology, target identification

## Abstract

The annual meeting “Signal Transduction—Receptors, Mediators and Genes” of the Signal Transduction Society (STS) is an interdisciplinary conference which is open to all scientists sharing a common interest in the elucidation of the signaling pathways mediating physiological or pathological processes in the health and disease of humans, animals, plants, fungi, prokaryotes, and protists. The 24th meeting on signal transduction was held from 15 to 17 November 2021 in Weimar, Germany. As usual, keynote presentations by invited scientists introduced the respective workshops, and were followed by speakers chosen from the submitted abstracts. A special workshop focused on “Target Identification and Interaction”. Ample time was reserved for the discussion of the presented data during the workshops. Unfortunately, due to restrictions owing to the SARS-CoV-2 pandemic, the poster sessions—and thus intensive scientific discussions at the posters—were not possible. In this report, we provide a concise summary of the various workshops and further aspects of the scientific program.

## 1. Introduction

This year, the organizing committee of the Signal Transduction Society (STS) was faced with the ongoing SARS-CoV-2 pandemic, which had led to cancellation of the annual meeting on “Signal Transduction—Receptors, Mediators and Genes” in 2020. Applying an elaborate hygiene concept and featuring a limited number of participants, the STS decided to hold the 24th Meeting on “Signal Transduction—Receptors, Mediators and Genes” as an on-site meeting in Weimar, Germany, from 15 to 17 November 2021. This year, the meeting was organized by the Signal Transduction Society (STS) together with the signaling study groups of the German Societies for Pharmacology (DGP), Cell Biology (DGZ), Biochemistry and Molecular Biology (GBM), and Immunology (DGfI). The collaborative research center (SFB) 854 “Molecular Organization of Cellular Communication in the Immune System” (Magdeburg, DE) provided further scientific and financial support. A special workshop on “Target identification and interaction” was organized. The contributions of young scientists have always been an integral part of the meeting program. Thus, all of the poster contributions could be presented as concise five-minute short talks in the plenum in one of the “My Poster in a Nutshell” sessions.

## 2. Meeting Overview

The 2021 meeting was held in a new format, starting at noon on Monday and ending at noon on Wednesday, in order to ensure a timely but convenient arrival at and departure from the meeting. The workshops “Differentiation, Stress, and Death”, “Protein Interaction and Signaling”, “Immune Cell Signaling”, “Infection and Inflammation”, and “Tumor Cell Signaling” were opened by keynote speakers introducing the audience into the field by presenting their recent research. In this year’s meeting, the classical workshops were enriched by a special workshop on “Target Identification and Interaction”, which was opened by two keynote presentations. The keynote lectures were followed by short talks that were selected from the submitted abstracts by the respective workshop chair people. Because a “classical” poster exhibition was not possible due to the SARS-CoV-2 pandemic, all of the posters could be presented in the plenum as five-minute short talks in one of two “My Poster in a Nutshell” sessions.

### 2.1. Workshop on Differentiation, Stress, and Death

Wolfgang Schamel, from the BIOSS Centre for Biological Studies (Freiburg, Germany), demonstrated a new paradigm of the ways in which cells integrate extracellular signals. Using T cells and the T cell receptor (TCR) as model system, he focused his recent research on the kinetics of the TCR–ligand interaction, instead of solely considering static binding processes. A very well-suited method for these investigations is the use of optogenetics, a technique using proteins that alter their conformation upon exposure to light of a specific wavelength. For the human TCR, his group developed the opto-ligand-TCR system, which is based on the Phytochrom B (PhyB) photoreceptor from *Arabidopsis thaliana* [1,2]. When exposed to light of a 660-nm wavelength, PhyB tetramers reversibly alter their conformation from “OFF” to “ON” in such a way that tight binding to the PhyB interacting factor (PIF) is possible. In the opto-ligand-TCR system, the PIFs are coupled to the TCR, such that the clustering of the PIFs leads to the activation of TCR signaling. In this context, the light intensity is the major determinant of the ON–OFF cycling rate, and can hence be used to test different binding durations. The mathematical modelling of the optogenetically induced TCR signaling uncovered the half-life of ligand–TCR binding as a central determinant of downstream signaling. By means of this kinetic proofreading, low-affinity ligands with a short half-life are interpreted as system noise, whereas high-affinity ligands being receptor-bound for time periods longer than the kinetic proofreading time will forward the signal into the cell. Next, the Schamel group experimentally mimicked the TCR–ligand encounter by migrating T cells. For this purpose, the ligand binding times and pauses were varied, and the activities of several signaling pathways were analyzed. Strikingly, the temporal patterns of ligand binding selectively activated different intracellular signaling pathways. Apart from the known conformational bias of the ligand–receptor interaction, this “kinetic bias” adds another layer of complexity to receptor signaling.

### 2.2. Special Workshop on Target Identification and Interaction

The special workshop was opened by Paul Lieberman (Wistar Institute, Philadelphia, PA, USA), who talked about Epstein–Barr virus (EBV)-associated diseases and the development of new drugs. EBV is a ubiquitous herpesvirus, and around 90% of the global adult population are thought to be persistently infected. EBV is estimated to be the cause for around 1% of cancers, and is associated with a variety of human disorders, such as nasopharyngal carcinoma, Burkitt’s Lymphoma, and 10% of all gastric carcinoma [3]. Despite the strong link to cancer development from latent infections, there is no specific EBV treatment which is available. Tumor cells harbor latent viral genomes that persist in the cell in a chromatin-associated manner. For this, the viral protein EBNA1 is a critical factor, as it regulates viral replication and the survival of infected cells. Because EBNA1 is expressed in all tumor types, Paul Lieberman’s group decided to develop small-molecule inhibitors. From the over 2000 synthesized molecules, one candidate was found that met the standards required for testing in further clinical trials [4]. In addition, these studies found new EBNA1 activities that advanced the understanding of its function and might open new opportunities for drug development. 

The second keynote presentation focused on the NF-kB pathway, and the possibility of targeting this molecule in a cancer-selective manner. NF-kB is a transcription factor that coordinates many central pathways, such as the host defense of innate and adaptive immunity, as well as stress, injury, apoptosis and cell survival. Around 70% of all cancer types show an upregulation of NF-kB-related signalling, making the protein an interesting target for drug development. Guido Franzoso (Imperial College, London, UK) illustrated the different approaches that had been used to inhibit NF-kB or its upstream regulators. Whilst the inhibition of the upstream regulator, the IKK complex, caused overt systemic inflammation, the targeting of IkB by proteasome inhibitors was also not favorable, as it is not specific for the NF-kB pathway or cancer cells [5,6]. Guido Franzoso’s group therefore chose a different strategy in order to find an NF-kB-related inhibitor for the treatment of multiple myeloma, an uncurable plasma B cell lymphoma. In their studies, they identified the immediate-early gene “growth arrest and DNA damage 45B” (GADD45) as a novel transcriptional target of NF-kB [7]. Interestingly GADD45B is a driver of NF-kB-mediated anti-apoptotic actions, and GAD45B expression levels correlate with the aggressiveness of the disease [8]. Indeed, the silencing of GAD45B triggered apoptosis via MKK7. This helped them to discover a tripeptide (DTP3) that was able to interrupt the GAD45B interaction with the JNK kinase MKK7, ultimately causing MKK7-mediated cell death. DTP3 shows a low KD, potent therapeutic activity and a good pharmacodynamic response, such that the number of CD138-positive plasma B cells was decreased significantly and in a cancer-specific manner in a phase I/IIa clinical study [9,10]. 

### 2.3. Workshop on Protein Interaction and Signaling

Volker Dötsch (Goethe University, Frankfurt am Main, Germany) presented—in his keynote lecture entitled “Mechanism of genetic quality control in oocytes”—new data on the function of TAp63α, one isoform of p63, as a quality control factor in female oocytes. TAp63α shares a high sequence identity with the famous tumor suppressor p53, and is highly expressed in oocytes. Oocytes have evolved a unique quality control system that eliminates cells with mismatched chromosomes or failures in DNA repair. Moreover, DNA damage is a key inductor in oocyte death. Volker Dötsch nicely demonstrated that DNA double-strand breaks result in the phosphorylation and subsequent activation of TAp63α; thus, TAp63α activity must be tightly regulated in oocytes [11]. By using a combination of biochemical, structural, cell culture and mouse experiments, Dötsch’s group discovered that TAp63α is activated in a multistep phosphorylation process involving Casein kinase 1 (CK1) and Checkpoint kinase 2 (CHK2). The sequential phosphorylation converts the closed dimeric conformation and thus inactive p63 form into an open, active, tetrameric state. He presented an elaborated model of the way in which these conformational changes are mediated within the molecule, and highlighted the kinetics within this process. TAp63α is phosphorylated in a biphasic manner, with two rapid phosphorylation events and a third slow phosphorylation event. Mechanistically, the third step defines the level of DNA damage that is necessary for oocyte apoptosis by enabling cells with low damage to survive by the rapid degradation of the activated TAp63α [11,12]. The accurately resolved CK1/TAp63α substrate interaction, in which the product of one phosphorylation step acts as an inhibitor for the following step, describes a new a molecular mechanism and demonstrates that the kinetics of the phosphorylation and the conformational changes in p63 determine the oocyte fate after DNA damage [13,14]. Thus, infertility—which is often seen as a side effect of cancer treatment for women—can be directly linked to the oocyte elimination induced by p63 activation.

### 2.4. Workshop on Immune Cell Signaling

The keynote lectures in this workshop were given by Friederike Berberich-Siebelt (University Würzburg, Germany) and Dirk Brenner (Luxemburg, Luxemburg), and covered different aspects of T cell biology. Friederike Berberich-Siebelt presented data on the possibility of performing gene editing on primary T cells. The allogenic transplantation of immune cells supports a graft-versus-leukemia or malignoma reaction (GvL), in which the residual tumor cells remaining after therapy are recognized and killed by the transplanted lymphocytes. Graft-versus-host disease (GvHD) is an unwanted complication that is caused by incompatibilities between the human leukocyte antigens (HLAs) in which transplanted T lymphocytes react against the host’s organs. Therefore, it would be favorable to prevent GvHD while preserving GvL. Therefore, techniques to manipulate T cells during adoptive transfers without the need to create transgenic mice would be advantageous. In her talk, Friederike Berberich-Siebelt presented an easy and inexpensive CRISPR/Cas9-based method to perform gene editing in primary murine T cells [15]. Her data demonstrated that guide RNA nucleofection is sufficient for highly efficient gene editing in both activated and naïve CD3^+^ T cells derived from Cas9 transgenic mice, and she discussed the possibility of expanding this approach in a translational setting.

Depending on their activation state, T cells show different metabolic activities. While naïve T cells depend on oxidative phosphorylation, the Th1, The and Th17 subsets show a strong glycolytic activity which enables them to react to the inflammatory stimulus with an anabolic production of humoral mediators [16]. Dirk Brenner presented data on regulatory T cells that highlighted their dependence on oxidative phosphorylation and the resulting accumulation of mitochondrial ROS. His work showed that these cells have a protective mechanism in which the anti-oxidant glutathione restricts serine uptake, as serine suppressed the Treg phenotype through the inhibition of FoxP3 expression [17]. He discovered that the loss of glutathione through the ablation of the gene for glutamate cysteine ligase (*Gclc*) changed the uptake of serine through ASCT1. This eventually shifted the Treg/Th17 balance towards autoimmunity and increased the anti-tumor response caused by the dampened Treg activity. Because the restriction of serine uptake restored the Treg function in mice, it is possible that these insights may help us to develop new therapeutical strategies to modulate the Treg/Th17 balance in cancer and autoimmunity in the future. 

### 2.5. Workshop on Infection and Inflammation

The workshop on Infection and Inflammation started with a fascinating study on antiviral immunity in children and adults. Infections with SarsCoV2 show an age-related pattern of infectivity, and young children seem to be less affected and have a lower risk for severe disease in many cases. Therefore, Marco Binder (DKFZ, Heidelberg, Germany) and colleagues investigated nasal swabs from infected and uninfected children and adults in order to understand the mechanism that seemed to protect young children. Using a single cell analysis approach with the immune cells and epithelial cells of the nasal mucosa, they saw that the receptors for virus entry did not show differential expression patterns. However, the pattern recognition receptors—RIG-I, MDA5 and LGP2—that mediate intracellular virus detection by innate immune cells were expressed at higher levels in children. Ultimately, this leads to higher levels of type I Interferons (IFN) at all times, and an even more pronounced antiviral Interferon-stimulated gene response in the presence of SarsCoV2 [18].

### 2.6. Workshop on Tumor Cell Signaling

Gudula Schmidt (University Freiburg, Germany) opened the workshop on tumor cell signaling with a concise presentation of her research on how bacterial toxins may be used to modulate signaling via small GTPases, especially in cancer. The modulation of RhoGTPase signaling can heavily influence the migratory, metastactic and invasive phenotype of cancer cells. Schmidt’s group could demonstrate that cytotoxic necrotizing factors (CNF) can induce an invasive phenotype in the non-tumorigenic MCF10A breast epithelial cell line by activating RhoGTPases [19]. This effect could be reduced by the knockdown of RhoC. Furthermore, CNF treatment and RhoA/C overexpression were accompanied by the increased expression of COX-2 and GPRC5A, causing an increased invasiveness and a decreased proliferation, respectively. Specifically, the presence of GPRC5A led to less EGF-dependent signaling through the stabilization of the monomeric EGF receptor (EGFR), thus inhibiting EGFR dimerization and phosphorylation. Interestingly, the knockdown of GPRC5A also inhibited EGF-dependent proliferation, an effect caused by EGFR downregulation [20]. As a tool for the further investigation of Rho signaling, Gudula Schmidt introduced the *Photorhabdus luminescence* toxin complex (PTC), which consists of the subunits TcA, TcB and TcC. PTC can be used as tool for the delivery of numerous proteins, e.g., toxins, into cells. By its insertion into cellular membranes and forming pores at an acidic pH, pentameric TcA is key to the final translocation of the TcC components into the host cytosol. An important feature of this machinery is that the assembly of TcB and TcC forms a molecular cocoon for the C-terminal region of TcC [21]. The native TccC5 subunit is known to ADP-ribosylate RhoA at glutamine 63, causing the persistent activation of the GTPase [22]. By substituting the C-terminus of TccC5 with the C3bot from *Clostridium botulinum* or YopT toxin from *Yersinia enterocolitica*, the translocation machinery of Tc proteins could be re-designed to introduce a RhoA-inactivating toxin [23]. As another element in the PTC toolbox, Schmidt introduced the affibody technology for the modulation of Rho signaling. Affibodies directed against Ras and Raf were already described in the literature in 2010 [24]. The introduction of Ras/Raf affibodies as cargo into the chimeric PTC delivery system yielded a new powerful tool for the introduction of the affibodies into the target cells. Thus, the PTC-mediated delivery of affibodies might be an interesting alternative to the introduction of the Ras/Raf-directed affibody for research and even therapeutic purposes.

### 2.7. The Fostering of Early Career Researchers

In 2021, the STS grant committee chose five students (Yue Gao, Sevinc Sultanli, Dayoung Yu, Heidelberg; Bahareh Jooyeh, Giessen; Anna Katharina Riebisch, Bochum) to receive travel grants of 250 € each to allow their meeting attendance. The two Silver Sponsors of the meeting, Jackson ImmunoResearch and Agilent, sponsored one grant each (Yue Gao and Dayoung Yu, respectively). This year, posters were presented in the plenum in the “My Poster in a Nutshell” sessions as five-minute short talks. The sponsor Origene donated a 250 € award for the best short talk to Victoria Fuhr for her presentation “ScRNA-Seq tracks the transcriptomic alterations of a sensitive mantle cell lymphoma cell line across ibrutinib treatment”. After the short talk session, all of the posters’ presenters were able to make individual appointments with interested meeting attendees in the poster exhibition area. Based upon the short talks and the assessment of the printed posters in the poster exhibition area, the workshop chair people selected five award-winning posters out of the many contributions, which were awarded prizes to a total value of 750 € (First Prize—250 €: Cristina Maria Chiarolla from Würzburg; Second Prize—200 €: Bernd Bufe from Zweibrücken/Kaiserslautern; Third Prize—150 €: Hagen Bachmann from Witten/Herdecke; Fourth Prize—100 €: Sevinj Sultanli from Heidelberg; Fifth Prize—50 €: Miriam Kelm from Kiel).

The STS Science Award is offered as a reward for excellent research by an early career researcher of the STS. It was first introduced in 2005, and has become a regular element of the annual STS Meetings. In 2021, the STS Science Award was shared by Sushmita Chakraborty (AIMS, New Delhi, India) and Sjoerd van Wijk (Goethe University, Frankfurt, Germany). Sushmita Chakraborty presented recent data on the importance of the T cell regulatory factor OX40 for the treatment of Pulmonary Sarcoidosis, a disease characterized by excessive inflammation in the lung. Her data show that the blocking of the OX40 pathway could be a therapeutical strategy to rescue defective Treg activity and inhibit excessive inflammation [25]. Sjoerd van Wijk reported on his research on autophagy-dependent cell death in glioblastoma cells. His findings established new mechanistic links between ER stress and ER-phagy that improve our understanding of drug-induced ER stress and the selective activation of organelle-specific autophagy [26]. The award, with a total sum of 1500 €, was donated by the Signal Transduction Society.

## 3. The STS Honorary Medal Award

The STS Honorary Medal was introduced in 2010 in order to honor outstanding scientists in the field of signal transduction. The previous winners of the award are Anthony Pawson, Tony Hunter, Carl-Henrik Heldin, Klaus Rajewsky, Jules Hoffmann, Mina Bissell, Tak Wah Mak, Michael Reth, Karen Vousden and Fred Wittinghofer. Since 2017, the medal has been awarded by the STS in collaboration with the International Journal of Molecular Sciences (IJMS). In 2020, the STS awarded the prize to Prof. Dr. Peter H. Krammer (Heidelberg, DE), but due to the SARS-CoV-2 pandemic no adequate medal ceremony was possible. Thus, the STS council and advisory board decided to postpone the festive award ceremony to 2021. Peter H. Krammer received the STS Honorary Medal Award for his lifetime contributions to the elucidation of cell death signaling. His ideas and his innovative experimental approaches exceptionally influenced our current understanding of apoptosis signaling. Professor Krammer has not only inspired but also supported and guided generations of students and fellows. He and his research impacted an entire scientific era.

The award ceremony was opened by a personal laudation for the awardee by Ingo Schmitz, a former PhD student in the Krammer lab, and now a Professor at the Ruhr University Bochum. The laudation was followed by the festive presentation of the medal by the STS council. The medalist then gave the “Honorary Medal Lecture”, in which he presented an overview of his research. He identified the death receptor CD95 (APO-1/Fas), the first known antigen to induce cell death, and demonstrated that apoptosis is an intrinsic physiological process within cells. Prof. Krammer characterized the death-inducing signaling complex and the function of its components, described activation-induced cell death (AICD) on the single-cell level, and showed how T cell death contributes to immunological tolerance. More recently, the Krammer group provided evidence for the annexin exposure of apoptotic cells serving a specific signal that induces a tolerogenic dendritic cell phenotype. The manipulation of this annexin-mediated immunosuppression might be a future therapeutic strategy in the treatment of cancers and immune disorders.

The lecture was followed by a discussion of Peter Krammer’s influential work on apoptotic signaling and its future implications.

## 4. Final Remarks

Despite the ongoing SARS-CoV-2 pandemic, the 24th STS meeting could be held due to the elaborate hygiene concept that was prepared by the organizers. Strict adherence to this concept made it possible to enjoy an interesting and stimulating meeting. The 25th “Silver Jubilee” STS Meeting will again take place at the Leonardo Hotel in Weimar from 2 to 4 November 2022. Preparations for the meeting have already started, and regular updates on the schedule and contents of the meeting can be found at https://www.sigtrans.de (accessed on 9 February 2022). Additionally, news regarding the work of the STS can be accessed via Facebook or through the Twitter account (@SignalSociety).

## Data Availability

Not applicable.
